# Implementing reablement for community dwelling people with dementia: A formative evaluation using single-case experimental design

**DOI:** 10.1177/14713012251323941

**Published:** 2025-02-25

**Authors:** Alexis Campbell, Christopher J Poulos, Caroline Takla, Joy Allen, Kylie Lemsing, Claire MC O’Connor

**Affiliations:** 7800 University of New South Wales, Sydney, NSW, Australia; Centre for Positive Ageing, 94268 HammondCare, Sydney, NSW, Australia; School of Population Health, 7800 University of New South Wales, Sydney, NSW, Australia; Centre for Positive Ageing, 94268 HammondCare, Sydney, NSW, Australia; Centre for Positive Ageing, 94268 HammondCare, Sydney, NSW, Australia; School of Psychology, 7800 University of New South Wales, Sydney, NSW, Australia; Neuroscience Research Australia, Sydney, NSW, Australia; Ageing Futures Institute, 7800 University of New South Wales, Sydney, NSW, Australia

**Keywords:** dementia, reablement, implementation, rehabilitation, functional ability, single case experimental design

## Abstract

**Background:**

Reablement is recommended to maximise functioning in people with dementia, yet in Australia, is not routinely available. This study aimed to provide insight into the implementation and program outcomes of reablement in real-world practice for a person living with dementia.

**Methods:**

Reablement was implemented for a client with dementia. In parallel, a formative mixed-methods pilot evaluation was performed, using single-case experimental A-B-A design (*n* = 1), supplemented by routinely collected pre-post program clinical measures. Implementation was evaluated qualitatively via clinical notes for fidelity, feasibility and client engagement.

**Results:**

Single-case experimental design outcomes indicated the program positively impacted the participant’s physical functioning. Additionally, most routinely collected pre-post clinical measures demonstrated improvement. Intervention fidelity varied, with differences in length and client engagement.

**Conclusion:**

Implementation of evidence-informed reablement has been shown to be feasible in real-world practice for a community-dwelling person living with dementia. Larger implementation trials are needed to build on preliminary outcomes to ultimately improve access to these important programs.

## Introduction

Dementia is one of the leading causes of disease burden and disability in Australia (Australian Institute of Health and Welfare, 2022) and worldwide (World Health Organisation, 2021). People with dementia experience progressive loss of functional ability necessitating increasing support to complete activities of daily living (Giebel et al., 2014; Liu-Seifert et al., 2015; Martyr & Clare, 2012). Functional ability is determined by the combination of an individual’s intrinsic capacity (the composite of their physical and mental capacities) and their environment (World Health Organisation, 2015). Accordingly, it is one of the leading determinants of quality of life (QoL) for people with dementia (Burks et al., 2021). Consequently, interventions that delay functional decline are highly valued by people with dementia (Guideline Adaptation Committee, 2016), particularly as pharmacological interventions offer limited efficacy in improving outcomes or slowing disease progression (Szeto & Lewis, 2016; Tisher & Salardini, 2019).

Healthcare systems are under pressure to provide quality dementia care services to a growing population using limited resources (World Health Organisation, 2017). Functional ability and length of admission to residential care (largely determined by functional ability) (Olsen et al., 2016) are the most important cost drivers in dementia care (Brown et al., 2017; Gustavsson et al., 2011; Standfield et al., 2019). At an individual level, transition to residential care is also predictive of significant negative changes in QoL (Olsen et al., 2016). Therefore, interventions that maintain function to prolong people with dementia living at home are desirable from both person-centred and economic view-points (Standfield et al., 2019; Tisher & Salardini, 2019).

Reablement is an emerging multidisciplinary approach that uses an individual’s intrinsic capacity and environmental modifiers (e.g. housing modifications, mobility aids and assistive technologies) to maintain function, regain lost function where possible and compensate for changes in function (Poulos et al., 2017). By maximising functional ability, reablement aims to increase QoL, reduce service utilisation (Tessier et al., 2016), decrease carer burden, and potentially delay the need for long-term residential care (Poulos et al., 2017). Reablement aligns with the broad recommendations for a wellness approach to care stated in the Australian Clinical Practice Guidelines for People with dementia (Guideline Adaptation Committee, 2016). However, a lack of clinical detail within the guidelines (for example, recommended intervention length, frequency and structure) mean challenges remain around delivering reablement in practice. A recent study of the Australian aged care sector found that reablement has thus far been inconsistently defined in the literature, leading to potentially improper implementation. As providers urgently offer interventions labelled ‘reablement’ to align with funding schemes, services delivered are not necessarily evidence-informed (O'Connor et al., 2020). To address this gap, a series of evidence-informed, freely available ‘reablement in dementia’ resources were developed (O'Connor, Gresham, et al., 2022; O'Connor, Rowlands, & Poulos, 2022) to support health professionals in designing, delivering and evaluating reablement programs for clients with dementia (Gresham et al., 2019; O'Connor et al., 2019; O’Connor & Poulos, 2021; Poulos et al., 2019).

It is widely acknowledged that successful implementation of evidence into clinical practice is challenging as research and clinical environments can be vastly different (Clemson et al., 2021; Culph et al., 2021; Proctor et al., 2009). Real-world clinical environments present a range of individual and system-wide factors that can impact the successful translation of research evidence (Azim et al., 2022). Studies to test the effectiveness of the translated research are needed to aid health professionals and potential clients in their decision making pertaining to funding, delivering or participating in these programs (O'Connor, Gresham, et al., 2022; Poulos et al., 2018). While the reablement in dementia resources have been freely available since 2018, there are yet to be any implementation studies of their use, and there is presently limited uptake of these resources within the Australian aged care sector (O'Connor et al., 2020; O'Connor, Gresham, et al., 2022). Greater understanding of the implementation of these resources within real-world practice, using existing sustainable funding sources is needed to help bridge the evidence-practice gap. The outcomes of these evaluations have the potential to inform future practice (Highfield et al., 2015), increase sustainability and drive uptake of evidence-informed reablement in the broader dementia care sector (Proctor et al., 2009).

Whilst large scale implementation trials are recognised as important, the quality of many implementation trials over more than a decade has been criticised (Lewis et al., 2018; Powell et al., 2012, 2015) and as such it has been proposed that pilot studies should play a greater role in preceding definitive studies in this area (Pearson et al., 2020). The role of a formative pilot study in an implementation context should extend to identifying potential causal mechanisms of changes to the research evidence in clinical practice, and facilitate an iterative process of refining intervention strategies and optimising their impact (Pearson et al., 2020). Pragmatic implementation-effectiveness studies have already been used in a similar Australian context to aid the translation of other evidence-based reablement programs (Clemson et al., 2021; Jeon et al., 2020).

Therefore, in advance of larger definitive implementation studies, this formative pilot study aimed to explore: (1) program outcomes for a community-dwelling person living with dementia who participated in a reablement program delivered by an existing allied health service, and (2) implementation of the ‘reablement in dementia’ resources, mainly the reablement handbook, within routine clinical practice.

## Materials and methods

### Design

**Figure 1.**
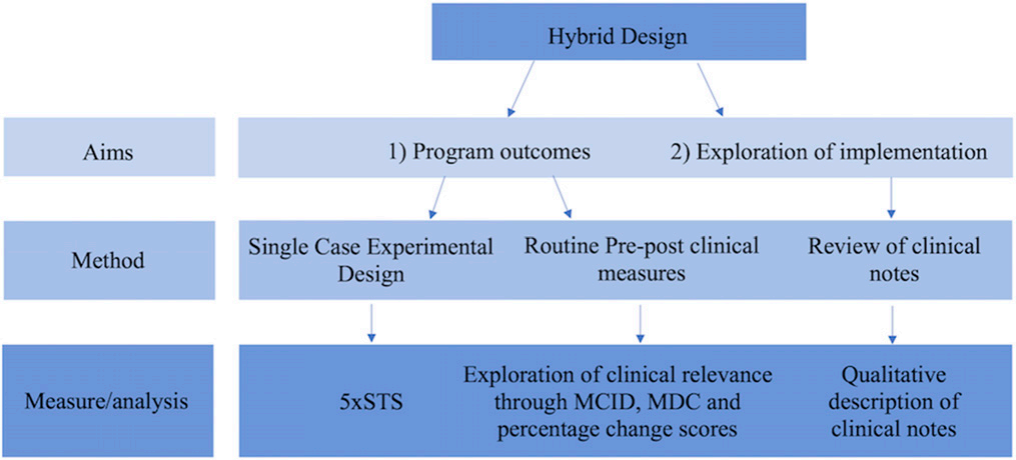
Overview of the mixed-methodology used within this study. 5×STS = Five Times Sit to Stand Test; MCID = Minimal clinically important difference; MDC = Minimal detectable change. See Table 1 for a more comprehensive list of outcome measures. Figure 1 Alt Text: A multi-level flow chart illustrating the hybrid design of the study. The first level shows that aims pertain to both program outcomes and implementation exploration. The second level shows methodologies used; specifically that exploration of program outcomes involved single case experimental design (SCED) and routine pre-post clinical measures, and exploration of implementation outcomes involved review of clinical notes. The final level shows measures and analysis processes used, involving 5xSTS for SCED, change scores and clinical differences for pre-post measures, and qualitative description of clinical notes for implementation exploration.

To address the dual research foci above, this study used a hybrid type 2 design with mixed methodology (Curran et al., 2012) to undertake a formative pilot evaluation (Stetler et al., 2006) of a reablement program designed using the reablement handbook (Poulos et al., 2019), delivered within an existing community aged care service setting. A simplified overview of the study design can be seen in Figure 1**.**

To add methodological strength to this study, a single-case experimental design (SCED) of A^1^BA^2^ withdrawal/reversal type was superimposed on the reablement program using a proxy outcome measure for functional change (Tate et al., 2016). In the baseline (A^1^) phase and the post-intervention (A^2^) phase the participant maintained their daily activities as usual. During the intervention (B) phase, the allied health team delivered a reablement program over eight weeks. The A^1^ and A^2^ phases lasted approximately two weeks, with A^1^ phase length determined by the time elapsed between program referral, study recruitment and reablement program start; A^2^ phase mirrored A^1^ in terms of both length and the number of datapoints collected. There was no washout period between the A and B phases.

The SCED methodology was designed according to the Risk of Bias in N-of-1 Trials Scale (Tate et al., 2013). In addition, routinely collected pre-post program clinical data were collated and qualitative clinical notes pertaining to program delivery and implementation were extracted (Kim et al., 2017).

### Setting

This study was conducted via an established community aged care service in Sydney, Australia. The research team worked collaboratively with the allied health team within the service to coordinate recruitment and data collection in parallel with routine service provision. The reablement intervention was delivered as part of a short-term restorative care program (STRC) (Australian Government, 2024). STRC is a federal government funded program for community-dwelling people over 65-years-old, which aims to improve functional ability to promote independence (Australian Government, 2024). Intervention delivery and outcome measurement collection were undertaken at times of mutual convenience within the participant’s home.

### Participant

Purposive sampling was undertaken to recruit the participant. Inclusion criteria were that the participant must: (a) have a diagnosis of dementia or identified memory impairment, (b) be living in the community and enrolled to receive a reablement program, (c) have capacity to provide informed consent or, if not, have a responsible guardian willing to provide consent on their behalf and support their participation, and (d) be 18-years-old or above.

### Ethics

Study approval was obtained from the University of New South Wales Human Research Ethics Committee (HC210759), and written informed consent was obtained from the participant.

### Intervention

During the B phase, the participant engaged in a reablement program based on the ‘reablement in dementia’ handbook (Poulos et al., 2019), tailored specifically to address their identified therapeutic goals. To promote fidelity to the resources in delivery of the reablement program, a virtual training session was provided to the allied health team by one of the researchers who was involved in development of the resources (last author). The reablement program was designed by the clinical allied health team (an occupational therapist and physiotherapist) in reference to the reablement handbook, but accounting for any restrictions of their clinical environment. For example, program length (8 weeks) was determined by the STRC funding structure. At the beginning of their program, the participant identified two primary goals for their program in collaboration with the treating occupational therapist and physiotherapist. The therapist-adaptation of these goals (O’Connor & Poulos, 2021) were:

Mobilise safely outdoors with a mobility aid for approximately 60 metres. (physiotherapist)

Increase safety and independence within the home using small aids for showering provided through the STRC program. (occupational therapist)

Based on these goals, program 4.1 (“supporting mobility and physical function through a falls prevention program” (p.38; Poulos et al., 2019) from the reablement handbook was used to inform the program that was delivered.

The occupational therapist assessed the participant’s functional ability, prescribed equipment to increase home safety (including a personal response system, a stair rail for home access and bathroom aids), and coordinated the participant’s care throughout the program. In parallel with the occupational therapist, the physiotherapist also performed an independent assessment of physical functional ability, delivered a series of face-to-face exercise sessions tailored to the participant’s physical and cognitive abilities, and prescribed a home exercise program to be completed outside these visits using physiotherapist-provided equipment (e.g. resistance bands and a pedal exerciser). A more in-depth exploration of the fidelity of the delivered program to the reablement handbook is included in the results section.

As part of the broader STRC program, through which the reablement intervention was being delivered, the participant received two in-person consults from a dietitian and podiatrist and one in-person consult from an audiologist.

### Outcome measurements

The SCED outcome measure was the Five Times Sit to Stand (5xSTS) (Bohannon, 2011). The 5xSTS has excellent reliability in demonstrating physical functional ability and falls risk in community dwelling older people (coupled with ease of administration and low cost), therefore the clinical allied health team deemed it an adequate proxy for the participant’s reablement goals (Buatois et al., 2008; Melo et al., 2019). The 5xSTS involves measuring the time taken for the participant to transfer five times from a seated position to a standing position as quickly as possible using a standard height chair (43–45 cm) with straight back (Melo et al., 2019). The less time taken to complete the test, the better the outcome. Performance times greater than 13.6 seconds are associated with increased disability and morbidity in community dwelling older adults (Ardali et al., 2020). As per SCED guidelines (Tate et al., 2016), a minimum of five 5xSTS data points were collected per phase. The proxy outcome measure was collected by members of the research team (A-phases; AC and CMCOC) and the clinical allied health team (B-phase).

Routinely collected pre-post clinical outcomes included a range of measures to capture personal goal attainment, quality of life, activities of daily living, nutrition, and physical functioning. Implementation outcomes included qualitative analysis of clinical notes to explore program fidelity to the reablement handbook, participant engagement with the program and whether or not there were adverse events associated with the program (Kim et al., 2017). Table 1 provides a detailed list of the included outcome measures.

**Table 1.** Outcome measures assessed in this study.

Relevant aim	Methodological approach	Outcome Measures/Analysis
1	Single case experimental design (SCED)	Five times sit to stand test (5×STS) (Goldberg et al., 2012; Melo et al., 2019) - Client specific measure of functional ability chosen by the treating physiotherapist.
	Routinely collected pre-post clinical measures	Goal attainment scaling-light (GAS-light) (O’Connor & Poulos, 2021; Turner-Stokes et al., 2009) - GAS-light is a method of recording and evaluating meaningful program outcomes to determine whether the client’s individual goals have been attained at the end of the program. - The functional SMART goal(s) are identified through collaboration between the client (and their family where appropriate) and the allied health team. At baseline, the client is rated as having has either some function (−1) or no function at all (−2) towards the goal. - At the end of the program the client’s goal is revisited to “determine whether the goal was achieved as expected (0), a little more than expected (1), a lot more than expected (2), or if it was not achieved, whether it was partially achieved or no change (−1) or if it got worse (−2)”(p.18) (O’Connor & Poulos, 2021). - Any changes to the score between baseline and program end are monitored.
		PROMIS-10 global health measure T-scores (Hays et al., 2009) - Client reported health status broken down into physical and mental raw scores. - Raw scores are converted to T scores which compare the client’s self-reported health to the United States general population such that a score of 50 represents the average. - A higher score indicates better client perception of health status.
		Modified Barthel index (Shah et al., 1989) - Clinician administered screening tool of client’s ability to complete activities of daily living with or without assistance. - A higher score indicates reduced dependence on external support to complete ADLs.
		Mini nutritional assessment short form (Kaiser et al., 2009) - Clinician administered screening tool for nutritional status. - A higher score indicates better nutritional status.
		Clinical frailty scale (Church et al., 2020) - Clinician administered screening tool for frailty. - Scored from 1-9 where 1 = very fit and 9 = terminally ill.
		Timed up and go (Christopher et al., 2021) - A timed clinical assessment of balance and general mobility. - A time above the cut off of 13.6 seconds is a predictor of falls risk in community dwelling older persons (Barry et al., 2014)
		Balance measures - Left and right timed tandem stance test and left and right single leg stance test from the Berg Balance scale (Kornetti et al., 2004) - All tests performed with bare feet. - Useful clinical indicator of falls risk in older adults.
2	Analysis of clinical notes to answer the following questions	Feasibility - Was the reablement program able to be delivered using existing funding models accessible to someone with dementia living in the community? - What were challenges/facilitators to reablement program delivery perceived by the clinical team?
		Fidelity - How closely did the reablement program delivered reflect the reablement handbook’s evidence-informed recommendations?
		Client engagement - What were practitioner perceptions regarding the level of client engagement with the program?
		Adverse events - Were any adverse events or safety issues reported?

PROMIS = Patient-Reported Outcomes Measurement Information System.

SMART = Specific, Measurable, Attainable, Relevant and Time-bound.

### Data analysis

The SCED outcome data was graphed for each stage and analysed visually as per recommendations for SCED studies (Kratochwill et al., 2010; Lane & Gast, 2014; Tate et al., 2016). Changes in mean, trend and variability of data across phases was used to evaluate the impact of the reablement program on the 5xSTS using systematised guidelines by Kratochwill et al. (Kratochwill et al., 2010) to increase the accuracy of interpretation (Krasny-Pacini & Evans, 2018; Kratochwill et al., 2010). In addition, percentage of non-overlapping data between phases was used to provide an indication of effect size (Chen et al., 2016).

For the routinely collected pre-post clinical measures, percentage change scores (baseline to post-program) were analysed to explore program outcomes. Percentage change scores were calculated for each outcome by dividing the change score (baseline score subtracted from the post-intervention score) by the absolute value of the baseline score, multiplied by 100 (Zhang & Han, 2009). Percentage change scores present results in clinically relevant terms to patients and clinicians and have been used previously in small sample studies (O'Connor et al., 2021; Savage et al., 2014). Where available, the authors report the meaningful clinical significance of change scores.

For the implementation data, clinical notes were content analysed to extract information around program feasibility, funding, fidelity, participant engagement, and whether or not there were adverse events associated with participating in the program. These details were compared to the reablement handbook (Poulos et al., 2019). No interviews were conducted with the participant, family or delivering service providers.

## Results

### Demographic information

Despite aiming for 3-6 participants to provide inter-subject replication, only one eligible participant was successfully recruited within the study timeframe. This participant completed their reablement program between June and August 2022.

The participant, NL (initials changed for anonymisation), was a 78-year-old female of culturally and linguistically diverse background with a primary diagnosis of Alzheimer’s disease. Further demographic and clinical characteristics are shown in Table 2.

**Table 2.** Demographic and clinical characteristics of the participant at baseline.

Participant, NL^a^	
Age, years	78
Sex	Female
Primary diagnosis	Alzheimer’s disease with concomitant vascular disease.
Comorbidities	Osteoarthritis, type 2 diabetes mellitus, hypercholesterolaemia, hypertension, osteoporosis, 2× stroke/transient ischaemic attacks reported in the last 12 months and bilateral total knee replacement.
Living arrangement	Lives with family, single storey home with steps at front and rear.
Baseline RUDAS score	20/30

RUDAS = The Rowland Universal Dementia Assessment Scale.

^a^Initials changed for anonymity.

NL lived in the community with her son’s family, was home alone a minimum of three days per week, and attended a day respite centre two days per week. The STRC referral reported that NL received minimal support from those she lives with apart from shopping and the preparation of evening meals. Whilst NL reportedly *“feels she can manage herself”* (dietitian notes), her healthcare was coordinated by her nephew who does not live with her but who *“expressed concerns regarding her memory and her ability to manage”* (Aged Care Assessment Team support plan). NL’s nephew was acting as her primary informal carer who *“phones daily and provides regular monitoring and prompts with medication and meals, transport to appointments and other tasks as required”* (Aged Care Assessment Team support plan). The physiotherapist identified that *“since the knee operation [year unknown] and moving houses [NL’s] mobility has declined”* (physiotherapist discharge summary).

### SCED outcomes

**Figure 2.**
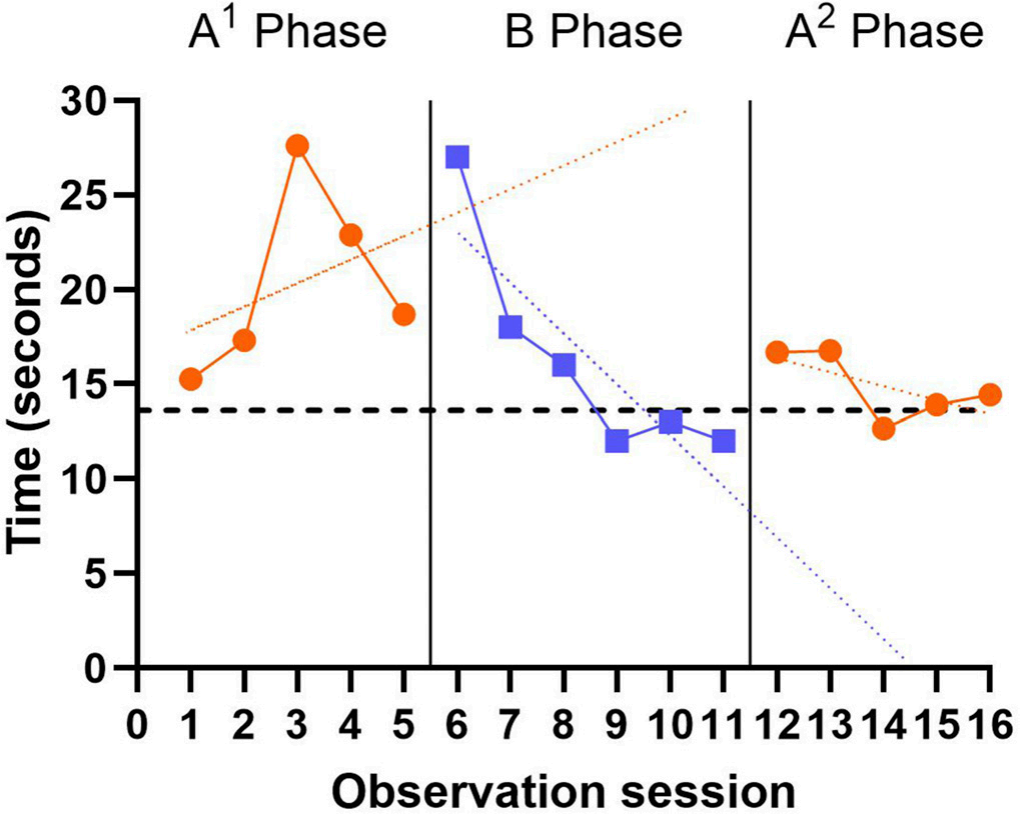
5×STS SCED outcomes across all three phases (A^1^BA^2^). A lower time indicates better performance. The dashed horizontal line at 13.6 seconds indicates the clinically significant cut-off over which there is an association with increased disability and morbidity in community dwelling older adults (Ardali et al., 2020). An extended celeration line has been added from A^1^ to B phase, and B to A^2^ Phase. A linear trend line has been added to A^2^ Phase for visual interpretation. Figure 2 Alt Text: three panel graph showing STS time points from each study phase. Panel one shows A^1^ phase times, panel 2 shows B phase times, and panel 3 shows A^2^ phase times.

Baseline A^1^ phase had a mean 5xSTS score of 20.4 seconds (standard deviation [SD] = 4.9 seconds). Visual inspection of the baseline phase suggested an increasing (i.e. worsening) trend across the phase with a clear pattern of all data points being above the clinically significant cut-off, indicating increased risk for disability and morbidity. During the intervention B phase, the mean 5xSTS score improved to 16.3 seconds (SD = 5.8 seconds), representing a 20% mean baseline reduction. This phase had a decreasing (improving) trend, with the latter three data points below the performance cut-off, indicating lower risk for disability and morbidity. When the intervention was removed in the A^2^ phase, the mean 5xSTS score further improved, decreasing to 14.9 seconds, with the narrowest variability of all the phases (SD = 1.8) and a decreasing (improving) trend (Figure 2). Reviewing the percentage of non-overlapping data, 50% (three data points) of the B phase were below the lowest A^1^ data points. When comparing B and A^2^ phases, there were no overlapping data. This indicates that while there was latency of effect between A^1^ and B phase initially, there was a sustained effect of intervention into A^2^ phase. In addition, there was a mean difference of 5.5 seconds between A^1^ and A^2^ phases, which is more than double the minimum detectable change identified for 5xSTS performance in community-dwelling females aged 60-years-old and above (Goldberg et al., 2012).

### Routinely collected clinical outcomes

Most routinely collected clinical outcomes improved from baseline to post-program (Table 3). Notable improvements were seen in the Timed Up and Go, which exceeded the minimum clinically important difference used in previous home-based exercise trials for older people (Clegg et al., 2014), and the PROMIS-10 Global health Physical T-score, which revealed the greatest improvement in percentage change. The clinical frailty scale and the Goal Attainment Scaling-Light scores (occupational therapist & physiotherapist) were the only measures not to undergo any change (Table 4).

**Table 3.** Outcomes of the routinely collected pre-post clinical measures.

Measure		Baseline	Post-Program	Percentage Change	Relevant Context
PROMIS-10 global health physical T-score		26.7	37.4	↑ 40.07% (improvement)	Score change crossed cut-point to move from ‘poor health’ to ‘fair health’ compared to the general US population (Hays et al., 2015).
PROMIS-10 global health mental T-score		31.3	41.1	↑ 31.51% (improvement)	Score change crossed cut-point to move from ‘fair health’ to ‘good health’ compared to the general US population (Hays et al., 2015).
Modified Barthel index (/100)		76	86	↑ 13.16% (improvement)	Score change did not meet minimal detectable change for the modified Barthel index of 15.4 (Yang et al., 2022).
Mini nutritional assessment (/30)		15	19	↑ 26.67% (improvement)	Score change from ‘malnourished’ to ‘at-risk’ of malnutrition (Vellas et al., 1999).
Clinical frailty scale (/9)		5	5	0 (no change)	No score change; remained ‘frail’ based on the cut-off score of ≥5 (Church et al., 2020).
Timed up and go (seconds)		16	13	↓ 18.75% (improvement)	Score change met the minimal clinically important difference of 1.4 seconds which has been used in a previous home-based exercise trial for older people with frailty (Clegg et al., 2014).
Single leg stance test (seconds)	Left leg	Unable	29	↑ Undefined (improvement)	Score change in left leg from severe mobility limitation to no limitation (Kim et al., 2009). Right leg remained in the severe limitation category.
	Right leg	Unable	3	↑ Undefined (improvement)	
Tandem stance balance test (seconds)	Left leg forward	2	27	↑ 1250% (improvement)	Score change in both legs from mobility categories of severe limitation to moderate limitation (left leg) and no limitation (right leg) (Kim et al., 2009).
	Right leg forward	2	43	↑ 2050% (improvement)	

PROMIS = Patient-Reported Outcomes Measurement Information System.

**Table 4.** Goal Attainment Scaling-Light scores.

Healthcare professional involved in goal setting	Client and therapist defined goal	Therapist adapted SMART goal	Baseline score	End of program score
Physiotherapist	“Address fear of falling”	“Mobilise safely outdoors with a mobility aid for approximately 60 metres”	−1 (some function)	−1 (no change)
Occupational therapist	“Stay safe and independent in my home”	“Increase safety and independence within the home using small aids for showering provided through the STRC program”	−1 (some function)	−1 (no change)

### Implementation outcomes

#### Feasibility and funding

The STRC package was a viable funding avenue for delivering an evidence-informed reablement program to a community-dwelling person with dementia. Feasibility of implementation was demonstrated by the collaborative approach exhibited by the occupational therapist and physiotherapist to supply equipment, assistive technology, education and tailored exercise sessions as per the reablement handbook.

#### Fidelity

Table 5 provides a summary of the reablement program that was delivered in comparison to program 4.1 from the reablement handbook (Poulos et al., 2019); features of both fidelity to and divergence from the handbook were identified. A noteworthy difference included the shortened duration of eight weeks (as opposed to the recommended 12) to fit within the confines of the STRC funding model. Another difference was around less frequent sessions per week, which was observed by therapists to be due to *“Lack of understanding and support from the carer/ family*” (physiotherapist). Care decisions and session coordination were done in consultation with NL’s nephew with whom *“multiple attempts to contact with no success [*via*]…email…voice messages….SMS”* (occupational therapist) were made from at least week 3 onwards. This meant that on two occasions NL missed planned in-person sessions as she was not home when presumed to be. The first physiotherapist session affected (week 5) was able to be rescheduled, however, the final planned physiotherapist (and STRC dietitian) sessions (week 8) were unable to be rescheduled due to the STRC program being completed. A follow up occupational therapy session in week five *“to assess for and discuss activity engagement”* (occupational therapist) was also unable to be performed. Overall, only one session per week was able to be delivered rather than the recommended two to three.

**Table 5.** Comparison of the reablement program delivered versus program 4.1 outlined in the ‘reablement in dementia’ handbook (Poulos et al., 2019).

Reablement handbook section 4: Supporting mobility and physical function through a falls prevention program 4.1 A multicomponent plan delivered at home		
	Recommendations extracted from the reablement handbook	Details of the reablement program delivered extracted from therapist notes
Duration	3 months	2 months
Session frequency (per week)	2–3	1 (8× physiotherapist and 1× allied health assistant visits in total)
Session length (minutes)	30–60	30–60
Members of the treating team	Physiotherapist Occupational therapist	Physiotherapist Occupational therapist Allied health assistant
Exercise	Individually tailored strength and balance exercises prescribed by a physiotherapist.	Exercise sessions tailored to NL’s mental and physical capacity focusing on: - Static Balance exercises, - Dynamic Balance exercises, - Lower limb strengthening exercises.
Home safety	An assessment of the home, followed by individualised recommendations to improve home safety, conducted by an occupational therapist or other suitable practitioner. At least 50% of identified home safety recommendations have to be implemented for the minimum requirements for benefit to be met. Provision of education on potential home hazards and how the environment impacts the person living with dementia.	Equipment provided by occupational therapist included: • Slip-resistant bath mat, • Long handled sponge, • Toe washer, • Padded steel shower stool, • Steel toilet surround height adjustable, • Bed rail, • Rail at front access of home. This equipment was determined to have not been used, possibly due to the lack of a follow-up occupational therapist visit meaning it was difficult for NL to *“understand the purpose of occupational therapy”* (occupational therapist). Physiotherapist provided education on: • Falls risks • Pain management
Technology	Assistive technology is implemented as required.	A personal response system was provided but it was deemed not to have been used.
Sustainability	A care worker or family member can be trained to deliver or support aspects of the program	Allied health assistant provided one supplementary exercise session before scheduling conflicts prevented further allied health assistant sessions. Attempts to involve the family were made but were unsuccessful during the program.

Importantly, handbook consistent practice was also noted, including session lengths of 30–60 minutes and the tailored nature of the exercises and home safety modifications that were delivered and/or prescribed. As per handbook recommendation, considerable attempts at increasing the sustainability of the reablement program through involving an allied health assistant and family were also made; these are discussed below.

#### Participant engagement

During face-to-face sessions, NL was able to *“engage in exercises with proper support and prompts”* (physiotherapist). However, it was surmised that outside of the face-to-face sessions NL was *“unlikely to complete her home exercise program independently due to apathy and memory issues”* (physiotherapist, discharge summary). This was potentially exacerbated by the above-mentioned lack of engagement from NL’s family. To support the participant in completing the exercises between visits when home alone and thus increase handbook fidelity of the program, the physiotherapist trialled visual aids, placed in clearly visible spaces in the home. These *“didn’t work well with NL as she doesn’t like things hanging on the wall/fridge”* (physiotherapist). Similarly, equipment provided by both the occupational therapist and physiotherapist was *“stored…in a cupboard to keep them neat and out of the way and [NL] would therefore forget to use them”* (occupational therapist).

Consequently, as it appeared NL needed social support to perform the exercises, an allied health assistant was engaged to assist with extra exercise sessions. However, they were only able to complete one session before scheduling conflicts meant that no further allied health assistant-led sessions were provided.

Another finding relevant to participant engagement was that NL *“continued to complain of isolation [and was] excited to have someone to talk to”* (physiotherapist) throughout the program. On multiple occasions therapist notes indicated the perception that NL received significant social benefits from the reablement program which were an additional benefit to the functional improvement aims of the reablement handbook.

#### Adverse events

No adverse events were reported for the duration of the program.

## Discussion

This study provides preliminary evidence regarding the feasibility of implementing evidence-informed reablement for community-dwelling people with dementia and the potential for positive program outcomes within a real-world clinical setting. There is a growing body of evidence for home-based reablement programs to enhance function and independence in community dwelling people with dementia (Cezar et al., 2021; Dawson et al., 2019; LeDoux et al., 2020; Teri et al., 2020; Ávila et al., 2018). However, most of these studies were performed in a research context. A reablement intervention has undergone implementation research in Australia, however this program requires specialised training of practitioners with associated financial investment (Clemson et al., 2021). To the best of the author’s knowledge, the current study is the first in Australia to specifically explore the implementation of, and program outcomes from, freely available, evidence-informed reablement resources in a real-world setting (O'Connor, Gresham, et al., 2022). Therefore, this study provides an important link between preliminary works that have been conducted within a research setting, and the translation of research evidence into practice, by continuing to bridge the reablement evidence-practice gap (Clemson et al., 2021; Corvol et al., 2018).

Positive outcomes were observed around both aims of this mixed-methods study, with reablement program benefits identified via the SCED and routinely collected clinical data, and feasibility of implementation of the reablement handbook demonstrated within an established community aged care service. Variable program fidelity to the reablement handbook was identified, often due to clinical constraints (Kourouche et al., 2021; McGee et al., 2018). This mirrors previous research, which has found that complex healthcare interventions are often not delivered as planned, as health professionals modify interventions to fit within their service context (Cross et al., 2022; French et al., 2021; Lorencatto et al., 2014; Toomey et al., 2015; Wenborn et al., 2021). Importantly, the reablement handbook itself acknowledges that the research evidence collated to form its recommendations are “often generated using strict protocols and specifically trained staff, and may be conducted within a research environment [meaning] that not all research protocols may be suitable to, or sustainable outside of, this environment” (p.9; Poulos et al., 2019). Therefore, despite the differences in program delivery that were reported, the positive quantitative outcomes observed for this participant suggest that pragmatic modifications to the ‘reablement in dementia’ resources to facilitate implementation within real-world practice may still generate positive outcomes for people with dementia, but further research is needed to determine the limits of this flexibility.

An important clinical challenge identified by this study was the difficulty of engaging the participant with dementia in their reablement program outside of face-to-face sessions with treating therapists. NL was identified by the physiotherapist as potentially being impacted by apathy which affects 50-70% of people with dementia (Selbæk et al., 2013). Apathy is defined as a loss of motivation that is accompanied by diminished self-initiated behaviour and reduced goal-directed cognitive activity (Tampi & Jeste, 2022). Consequently, it is unsurprising that NL did not participate in her between-session home exercise program, and it is likely that many others with dementia would face similar challenges as supported by previous work that proposes apathy creates barriers to goal pursuit in reablement (Golenko et al., 2022).

Considering these challenges with program engagement, informal carers such as family have long been established as integral to motivate the person with dementia and support them in activity initiation and engagement (Baber et al., 2021; Chang et al., 2021). Indeed, family carers are a vital determinant of positive health outcomes for people with dementia, providing a protective effect against the risk of transition to residential care, and a better quality of life (Farina et al., 2017; Olsen et al., 2016). As encouraged by the sustainability recommendations of the reablement handbook, the physiotherapist and occupational therapist endeavoured to engage the family as active members in the reablement program, though this was ultimately unsuccessful. Prior studies have noted that opinions and expectations of health professionals and patient families can often be mismatched in reablement programs (Hjelle et al., 2016; Jakobsen et al., 2019). This has previously been theorised to be due to insufficient clarity regarding the limits of involvement of next of kin, how the distribution of responsibility should be assigned between next of kin and health professionals, and what each party hopes the program will achieve at the start (Jakobsen et al., 2019). There is potential that such a mismatch between client, family and health professional expectations about the reablement program contributed to the communication challenges and consequent departures from fidelity (e.g. lack of occupational therapist in-person follow up) found in this study.

Clinicians should aim for a more family-centred approach that encourages greater dialogue between people with dementia, their next of kin and health professionals (Doh et al., 2020; Hjelle et al., 2016; Jakobsen et al., 2019). However, challenges exist around successfully engaging family in reablement programs. A recent study (Jakobsen et al., 2019) outlined the paradox that even when health professionals acknowledge next of kin as important partners in reablement, they are simultaneously unsure how to effectively collaborate, particularly in Western cultures where there is significant value placed on the autonomy of the client with dementia. Healthcare professionals must navigate maximising family involvement without exerting pressure on next of kin to fulfill a role beyond their capacity as this has been shown to have negative health consequences for this group (Jakobsen et al., 2019; Poulos et al., 2019). Clear front-end communication of program extent, objectives, and review appointments both with participants and their family may help to align program aims for each person, thus improving compliance with the planned intervention and avoiding some of the challenges encountered in the present study.

This formative pilot study faced considerable challenges recruiting participants as a result of the constraints associated with working with a singular service over a limited time-frame in a low incidence population, as previously recognised (Olive & Smith, 2005). Considerable literature highlights the difficulty research studies have meeting recruitment targets (Beckers et al., 2019; Duncan et al., 2018; Gorton et al., 2022; Realpe et al., 2021). Simultaneously, it is well documented that people with dementia are a hard-to-reach population and recruiting the numbers needed for research relevant to them is challenging (Bartlett et al., 2019; Black et al., 2014; Cooper et al., 2014). This is important as future definitive implementation studies will likely face similar challenges which should be considered in project design. A recent exploration by Field et al. (Field et al., 2019) of organisational and individual difficulties with research recruitment of people with dementia provided recommendations for future recruitment planning. These recommendations should be considered when designing future SCED based service evaluations to address the barriers faced in this project.

This study has some limitations that must be considered. As this study involved evaluation of implementation within routine clinical practice, it was not possible to provide extensive intra-case replication (e.g. ABAB design). We therefore aimed to provide inter-case replication by recruiting 3-6 participants to allow for the establishment of a multiple baseline design that would have increased replication and external validity (Krasny-Pacini & Evans, 2018; Kratochwill et al., 2010; Tate et al., 2016). Ultimately, challenges around recruiting within a clinical context led to a small sample size. Despite this, outcomes provide important preliminary evidence on the feasibility and potential positive impact of delivering reablement for people with dementia living in the community.

Despite the limitations of SCEDs, using recommended visual analysis procedures have been recognised as valuable in bridging the evidence-practice gap (Tate & Perdices, 2020). Whilst visual analysis has notable shortcomings, it is the most appropriate approach as applying inferential statistics to SCEDs, particularly single participant studies, is inappropriate as the data are inherently autocorrelated, thus limiting the choice of appropriate statistical analyses (Beeson & Robey, 2006; Olive & Smith, 2005). When compared to a pre-post design, the A^1^BA^2^ withdrawal/reversal design itself strengthens internal validity (Tate et al., 2016). While the A^1^BA^2^ design is not the highest standard of SCED (Krasny-Pacini & Evans, 2018), it was fit for purpose within this service context where only one intervention period (B) was attainable due to the financial limitations imposed by STRC funding, and therefore, provides greater applied clinical potential (Australian Government, 2024). Indeed, it has been argued, that in the age of personalised medicine, the restrictive inclusion criteria of gold-standard randomised control trials makes these difficult to generalise and translate into real-world practice (Krasny-Pacini & Evans, 2018).

As a formative pilot study, the findings presented here are only preliminary and should be interpreted with caution, however, through evaluation of handbook-recommended versus actual reablement implementation, study outcomes have potential to inform the design of more definitive implementation studies in the future and provide guidance to health professionals currently implementing the resources in practice to optimise their impact.

Recommendations for future research include further work into the flexibility of reablement resource implementation within a variety of service contexts and funding models. Replication via SCEDs can be achieved across studies as long as the 5-3-20 rule is satisfied (i.e. “a minimum of five SCED studies examining the intervention, conducted by at least three different research teams in three different geographical locations with a combined number of 20 single-cases across the papers”) (pg 21; Kratochwill et al., 2010). The present study used only one of 27 distinct ‘programs’ of the reablement handbook as this was relevant to the participant and their goals; it is likely future studies in other contexts will use other handbook subsections. Future studies therefore could have the twofold effect of extending the research to other ‘programs’ within the handbook and replicating evidence to strengthen generalisability of results regarding implementation of the ‘reablement in dementia’ resources. Finally, the authors would like to encourage research into the implementation of reablement in rural areas, particularly via telehealth to determine its unique challenges and outcomes for this underserviced population (Bauer et al., 2019; Sekhon et al., 2021).

## Conclusion

This study provides insight into how evidence-informed reablement can be implemented within clinical practice to support community dwelling people with dementia. Despite variable fidelity to the reablement handbook, improvements to the participant’s physical functional abilities were observed. Larger implementation trials are the vital next step to build on these preliminary positive findings. In addition, whilst this study reported outcomes from one specific ‘reablement in dementia’ program, future work should explore the feasibility of other sections of the ‘reablement in dementia’ resources in different contexts. It is hoped that healthcare professionals will consider the challenges outlined above when designing reablement programs, and also the potential for modifying programs to fit within real-world contexts. The benefits of engaging in evidence-informed reablement programs for people living with dementia should be a key focus in future service development. By demonstrating a financially feasible pathway the authors hope that future service providers will be empowered to identify similar funding avenues, and inspired to seek new ones to improve access to reablement for people with dementia living in the community.
